# Stability of gel wax based optical scattering phantoms

**DOI:** 10.1364/BOE.9.003495

**Published:** 2018-07-02

**Authors:** Charlotte J. Maughan Jones, Peter R. T. Munro

**Affiliations:** 1 Department of Medical Physics and Biomedical Engineering, Malet Place Engineering Building, University College London, London WC1E 6BT, UK; 2 School of Electrical, Electronic and Computer Engineering, The University of Western Australia, 35 Stirling Highway, Perth, Western Australia 6009, Australia

**Keywords:** (290.0290) Scattering, (290.4020) Mie theory, (170.3880) Medical and biological imaging, (160.4760) Optical properties, (120.5820) Scattering measurements

## Abstract

Phantoms with tuneable optical scattering properties are essential in the development and refinement of optical based imaging techniques. Mineral oil based ‘gel wax’ phantoms are the subject of increasing interest due to their ease and speed of manufacture, non-toxic nature, ability to cast into anatomically realistic shapes, as well as their cost-effective nature of production. The addition of scatterers such as titanium dioxide powder and monodisperse silica microspheres to the gel wax allows for the creation of phantoms with a controllable optical scattering coefficient. To enable repeated use of such phantoms, the stability of the scattering properties must be determined–a property which has yet to be investigated. We present an analysis of the stability of the reduced scattering coefficient ($\mu_{s}^{'}$) of such phantoms over time. We conclude that due to the measurable reduction in scattering coefficient over time, gel wax phantoms embedded with silica spheres may not be suitable for repeated use over time, however gel wax-TiO_2_ phantoms are much more temporally stable.

## 1. Introduction

Phantoms are essential for the development and refinement of biomedical imaging techniques, including many optical techniques. Multiple matrix materials have previously been used in such phantoms for optical applications [1,2]. Indeed, silicone and TiO_2_ powder phantoms are widely used in biomedical optics applications, both with and without additional absorbers. Such phantoms are considered stable and reproducible, and have previously been used in, for example, optical coherence tomography [3] and elastography [4], as well as to create complex multi-layered tissue mimicking phantoms [5] and in the creation of general optical phantoms for use in the near infrared [6]. However such silicone phantoms require long manufacturing processes [7], as described in Section 3.1. Epoxy resin is another example of a phantom matrix material which also requires a lengthy manufacturing process [3]. Agarose is yet another matrix material which, however, requires specialist additives and storage methods to increase its shelf life [8].

Gel wax, a mineral oil based, gel like, candle making material, has gained increasing interest as both an optical and acoustic phantom material [9–12] due to its wide availability, low cost and non-toxic nature. There is particular interest in using it as a tissue mimicking material for developing imaging phantoms for photoacoustic imaging [9]. It can be used within a 3D printing system [12], however, if the specialist printing equipment is not available, or cost effective, then a simple manufacturing method for phantoms composed of gel wax with embedded scatterers and absorbers has been presented [9,10]. As well as casting into arbitrary shapes [11], gel wax has already been successfully used to create a variety of anatomically accurate phantoms in conjunction with 3D printed molds of the heart and placenta [10]. Optical scattering and absorption have been modulated by the addition of titanium dioxide (TiO_2_), carbon black and colored inks [9], whilst acoustic properties have been controlled by addition of glass spheres and paraffin wax [10] as well as graphite and TiO_2_ powder [12].

Silica microspheres have not yet been used in gel wax, although silica powder has previously been used in a mineral oil based ultrasound phantom [13]. Silica microspheres are often used as scatterers in optical phantoms as they are able to be made highly spherical and in batches with very narrow diameter distributions. They also result in an anisotropy factor (*g*) of approximately 0.9 which is more tissue realistic than other inorganic scattering materials. Despite their high cost, and well documented predisposition to clumping due to charge interactions [7,14], silica microspheres continue to be a popular choice of optical scatterer as they are able to be modelled accurately using Mie and continuum theory [15].

TiO_2_ powder is a widely available and affordable scatterer and has already been adopted in gel wax phantoms [9,11,12]. However, their irregular shape and broad particle size distribution [16] means that scattering cannot be modelled with the same accuracy as silica microspheres.

It is highly desirable that the properties of phantoms remain constant over time to enable repeated use. Heterogeneous gel wax phantoms containing inclusions colored with ink have been reported to be stable over a time period of 1 year, however, the stability was determined on the basis of visual inspection and was thus subjective [9]. No studies have, to date, attempted to quantify the optical stability of gel wax phantoms. In this study we monitor the scattering properties of scattering-only optical phantoms composed of silica microspheres embedded in gel wax, as well as TiO_2_ powder embedded in gel wax over approximately 10 months after manufacture. By monitoring these properties, and with comparison to optically stable silicone and TiO_2_ phantoms, we are able to assess the suitability of such phantom mixtures for long term use. Ideally we would also have monitored the stability of phantoms made from silicone and silica microspheres. However, we were unable to produce such phantoms with a scattering coefficient high enough to be reliably retrieved, presumably as the refractive indices of the silicone and the silica microspheres are too similar.

## 2. Materials and methods

### 2.1 Phantom materials

Phantoms were constructed using a mineral oil based candle wax material known as gel wax (Mindsets (UK) Ltd., Saffron Walden, UK) as the matrix material. Optical scattering was introduced by embedding either monodisperse silica microspheres of 1µm diameter (Pinfire – Gems and Colloids, Frankfurt, Germany) with a coefficient of variance of <5%, or TiO_2_ powder of mean particle size <5µm (Product 224227, Titanium(IV) oxide, rutile, Sigma Aldrich, Dorset, UK). The manufacturers do not specify the nature of the distribution of TiO_2_ particles diameters, however, a recent publication found that the same product, albeit probably from a different batch to that employed in this study, had a diameter distribution of 734 ± 310 nm (mean ± standard deviation) [17]. The refractive index of the silica microspheres (n_silica_) was quoted as 1.467 at 589 nm by the manufacturer. The refractive indices of the TiO_2_ (n_TiO2_) and gel wax (n_gelwax_) were not provided by their manufacturers, therefore 2.5082 [18] and 1.4 at 589 nm were used, respectively. The value of n_gelwax_ = 1.4 was used as gel wax is composed of mineral oil, which is a mixture of alkanes. Refractive indices at 589 nm, for a variety of alkanes and their mixtures, fall broadly between 1.35 and 1.45 [19] and so 1.4 was used as an estimate. We note, however, that the precise value of the refractive index of gel wax is not required to support the conclusions of this paper.

### 2.2 Phantom manufacture method

**Fig. 1:**
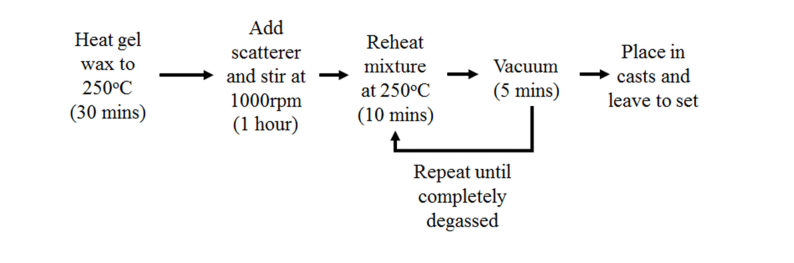
Outline of gel wax phantom manufacture.

The phantoms were manufactured using a modified version of the method presented by Maneas et al. [9,10] as outlined in Fig. 1

. The casts were manufactured from two soda lime glass slides, with two stacked coverslips (thickness 0.19 to 0.25 mm) at each end as spacers between the slides. As gel wax begins to set immediately on contact with a cool surface, the slides were heated to aid casting. This method of casting created phantoms of approximate thickness between 0.5mm to 1mm, specifically designed for use within the spectrophotometer (Perkin Elmer, Lambda 750, dual beam with 100mm single integrating sphere accessory). All phantoms were stored at room temperature for the duration throughout which the results reported in this manuscript were acquired.

### 2.3 Samples

Four concentrations of silica microspheres in gel wax were prepared, along with five concentrations of TiO_2_ powder in gel wax as outline in Table 1

**Table 1:** Summary of gel wax phantom properties

Matrix Material	Scatterer	Sample name	% scatterer by weight	Initial $\mu_{s}^{'}$ value (mm^−1^)
Gel Wax	Silica Microspheres	1A	7.32	0.54
		1B	14.65	1.13
		1C	21.94	1.34
		1D	29.42	1.57

	TiO_2_ powder	2A	0.18	0.44
		2B	0.36	0.95
		2C	0.53	1.30
		2D	0.71	1.78
		2E	0.89	2.26

.

**Fig. 2:**
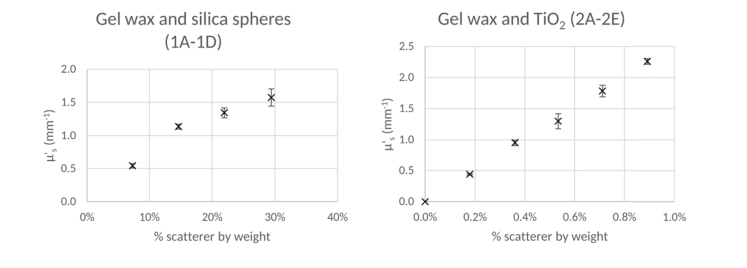
The relationship between scatterer concentration of and initial $\mu_{s}^{'}$ value calculated using the spectrophotometer for (left) gel wax and silica sphere and (right) gel wax and TiO_2_ powder phantoms.

The proportion of scatterer by weight was calculated from the initial mass of scatterer in relation to the initial mass of gel wax used to create each phantom. For each concentration, 4 separate phantoms were made, each of which was measured via the spectrophotometer once, with the mean of these measurements used to determine the $\mu_{s}^{'}$ value of each batch, which is the value considered from here on, as well as in Table 1 and Fig. 2

. The plots in Fig. 2 show that the gel wax with TiO_2_ powder exhibits a highly linear relationship between $\mu_{s}^{'}$ and percentage of scatterer, by weight, whereas the gel wax with silica microspheres is only approximately linear. One reason for this may be that at higher scatterer concentrations such as are present in the silica microsphere phantoms, $\mu_{s}^{'}$ can be reduced due to concentration dependent scattering [20]. Another explanation could be the aggregation of silica microspheres which we expect to be more prominent as scatterer concentration increases.

### 2.4 Optical property measurement

The values of $\mu_{s}^{'}$ were determined periodically, over the course of approximately 10 months, using a dual beam spectrophotometer along with the inverse adding doubling programme (IAD) [21,22]. The reflectance (%R) and transmittance (%T) measurements for each sample, along with the correction measurements, were performed in line with recommendations in the IAD handbook [22]. %R and %T were measured, and therefore $\mu_{s}^{'}$ was determined at 589 nm due to the value of n_spheres_ being provided by the manufacturer solely at this wavelength, and therefore the IAD parameters used were considered more accurate at this wavelength compared to others. As input to IAD, the anisotropy factor (*g*) for gel wax and silica sphere phantoms was estimated as 0.9, and 0.7 for gel wax and TiO_2_ powder based on the refractive index values previous stated, along with the particle size stated by the manufacturers. Furthermore, the refractive index of all phantoms was estimated as being that of plain gel wax. We note also that dual beam corrections were applied.

## 3. Results

### 3.1 Silicone and TiO_2_ powder

The silicone (Elastosil RT 601 A/B – Wacker Chemie AG., Munich, Germany) and TiO_2_ phantoms were manufactured using a previously developed method for silicone and silica microspheres [7] which we describe here briefly. The method begins by adding the required mass of TiO_2_ scatterers to the required mass of silicone part A. These two masses are calculated to achieve a design concentration of scatterer by weight. However, the final concentration (1.4% in this study) is calculated using the weighed masses, rather than the design masses, which may vary due to experimental limitations. Hexane is then added to the mixture at a volume ratio of 1:1 with the silicone part A. The mixture is mechanically stirred for an hour, placed in an ultrasound bath for an hour and then placed in a vacuum chamber for two hours to remove air bubbles. The mixture is then placed in the ultrasound bath for a further hour. The silicone part B is then added at a ratio of 9:1 (A:B), and it is assumed that the hexane has completely evaporated by this stage. The mixture is then gently stirred for ten minutes, to avoid the introduction of air bubbles, before placing the mixture into the vacuum chamber for ten minutes. The mixture is then poured into casts made of microscope slides and cover slips as described in section 2.2. However, in the case of silicone phantoms, it was necessary to heat the microscope slides to above 200°C and then allowed to cool, to prevent the phantoms from adhering to the surface. Once poured into the cast, the phantoms were cured at 70°C before being removed from their casts

**Fig. 3:**
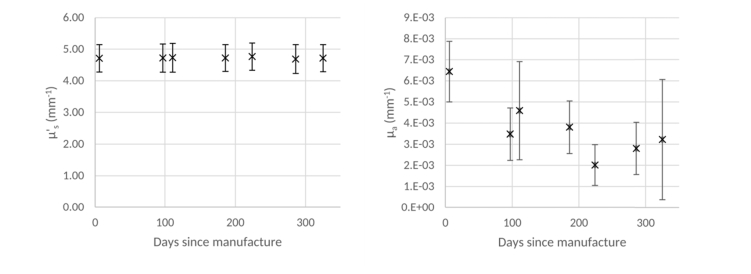
$\mu_{s}^{'}$ (left) and *µ_a_* (right) values of silicone and TiO_2_ powder phantoms, at a wavelength of 589 nm, over time. Error bars show +/− 1 standard deviation

The silicone had a refractive index of 1.409 at 589 nm which was obtained from the manufacturer, and the value of *g* for the combination of silicone and TiO_2_ particles was estimated to be 0.7. Figure 3

shows the high temporal stability of $\mu_{s}^{'}$ for these phantoms over a prolonged time. This data was obtained using dual beam spectrophotometer along with the IAD program as described in the previous section. Due to the high temporal stability of optical scattering in these phantoms we consider them the gold standard for this study. In particular, we have used them as a reference to ensure the consistency of all other measurements made within this study. Figure 3 also contains a plot of the distribution of absorption coefficients, $\mu_{a}$, retrieved by the IAD program, which shows that the silicone has a low absorption coefficient, as expected. The stability of the scattering also means that these phantoms can be used as a control for the measurement technique, with significant changes in $\mu_{s}^{'}$ being attributed to measurement error as opposed to phantom instability.

### 3.2 Gel wax and silica spheres

Fig. 4

**Fig. 4:**
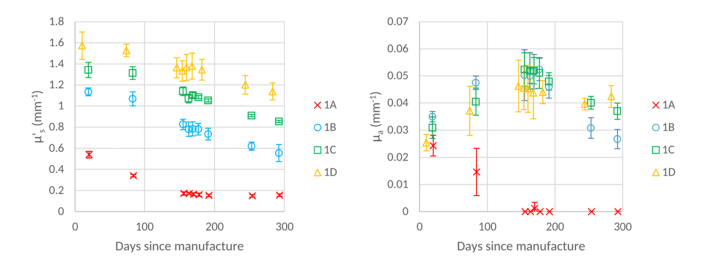
$\mu_{s}^{'}$ (left) and *µ_a_* (right) values of gel wax and silica microsphere phantoms, at a wavelength of 589 nm, over time. Error bars show +/− 1 standard deviation.

shows that $\mu_{s}^{'}$ decreases over the 10 month period studied for all gel wax and silica microsphere phantoms, regardless of their scatterer concentration. It can be observed that the scattering coefficient of the phantom with the lowest concentration of spheres (1A) plateaued after approximately 150 days post manufacture, however the final $\mu_{s}^{'}$ value is less than half that of the initial value. All other phantoms with higher sphere concentrations (1B, 1C, 1D) continue to decrease, with (for example) the measured $\mu_{s}^{'}$ value of 1B decreasing to approximately 50% of its initial measurement over a 10-month period. On visual inspection there are no obvious signs of degradation such as, for example, discolouration. Whilst clumping is visible after 10 months, this is a subjective observation. For reference we have included corresponding plots of $\mu_{a}$ which highlight the low absorption of gel wax, which is nonetheless higher than silicone.

### 3.3 Gel wax and TiO2 powder

Figure 5

**Fig. 5:**
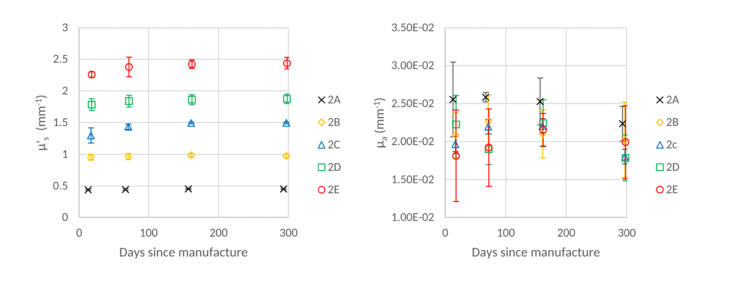
$\mu_{s}^{'}$ (left) and $\mu_{a}^{'}$ (right) values of gel wax and TiO_2_ microsphere phantoms, at a wavelength of 589 nm, over time. Error bars show +/− 1 standard deviation.

shows that the $\mu_{s}^{'}$ values for gel wax and TiO_2_ phantoms remain stable for phantoms of all scatterer concentrations over the time period measured. There are observable differences amongst measurements performed on the same sample. These differences are small and are mostly within the uncertainty (+/− 1 standard deviation) of the other measurements. Despite this, the phantoms are not as stable as the silicone and TiO_2_ microsphere phantoms previously discussed. We also note that $\mu_{s}^{'}$ appears to increase with time in Fig. 5, especially in the first 60 days. However, only in the case of the highest concentration is this increase outside the measurement uncertainty. In particular, for the highest concentration, there is an increase in the value of $\mu_{s}^{'}$, beyond the measurement uncertainty between the initial and final measurement. Given the stability of the lower concentration phantoms, we believe that this is most likely to be the result of a systematic error not accounted for by our uncertainty estimate. The associated plot of $\mu_{a}$ shows that, within the uncertainty of the retrieved values, the value of $\mu_{a}$ remains constant over time.

## 4. Discussion

The structure and precise chemical composition of gel wax is unknown. However, it is known to be composed of a mineral oil (e.g. paraffin oil) along with polymer resin, which allows the, normally liquid oil to set into a soft gel material. It is therefore proposed that the mechanism by which $\mu_{s}^{'}$ reduces over time in the gel wax and silica microsphere phantoms is the non-polar, semi-solid/gel like nature of the gel wax allowing the charged silica spheres to migrate through the matrix. Silica spheres have a predisposition to clumping due to electrostatic charge interactions between them [14]. When held in a solid matrix such as silicone, the particles are unable to move, and remain homogenously dispersed. However, when the viscosity of the mixture if reduced (as with gel wax), the spheres can slowly move towards each other, and this aggregation therefore increases over time.

There are other phenomena such as settling due to gravity which may explain the presented results. However, the tabulated densities of silica and TiO_2_ are 2.648 and 4.23 g/cm^3^, respectively [23]. Using mean diameter values of 1 and 0.734 μm, for silica and TiO_2_ microspheres, respectively (see section 2.1) the mean masses of the microspheres are given by 1.4 × 10^−18^ and 0.88 × 10^−18^ g, respectively. This means that the silica microspheres experience a force, due to the earth’s gravitational pull, 1.6 times greater than the TiO_2_ microspheres. However, given that the mean cross-sectional area of the silica microspheres is 1.9 times that of the TiO_2_ microspheres, we don’t expect gravitational settling to be more significant for the silica microspheres compared with the TiO_2_ microspheres. Furthermore, all phantoms were stored in the same upright manner, mounted between two microscope slides, with their thinnest edge aligned vertically. Thus, if there were significant gravitational settling in the phantoms containing silica microspheres, we would expect comparable gravitational settling in the phantoms made of gel wax and TiO_2_ microspheres.

We note that this work is limited to thin samples as are required for analysis by the spectrophotometer. It would interesting in future work to consider whether the phenomenon reported in this manuscript occurs for thick samples also. This, however, will be difficult to quantify due to the lack of reliable methods for making spatially resolved measurements of the scattering coefficient. Despite this, we are unable to observe by visual inspection any scatterer concentration gradient in thick phantoms that we have produced for other purposes.

The phantom with the lowest concentration of spheres reached a plateau with its $\mu_{s}^{'}$ value after approximately 150 days. This believe this is because, as the concentration of spheres increases, the likelihood of aggregation also increases since the spheres are much closer to each other, and therefore, are more likely to undergo a charge interaction and aggregate. It would be expected that, eventually, aggregation will reach a maximum due to the distance between neighbouring spheres increasing leading to the value of $\mu_{s}^{'}$ plateauing for all the sphere concentrations.

Titanium dioxide particles tend towards aggregation due to Van der Waals forces [24] that can form between them when they come into close proximity. The percentage of scatterer by weight values for the TiO_2_ phantoms are all below 1%. The distance between the TiO_2_ particles means that the likelihood of such Van der Waals interactions and, therefore, aggregation is low. If the concentration of TiO_2_ were to be significantly increased, then a similar decrease in $\mu_{s}^{'}$ may possibly be observed, however, at concentrations required to obtain tissue realistic scattering coefficients, they appear to be temporally stable.

Due to the instability of scattering properties in the gel wax and silica sphere phantoms, if the same gel wax and silica microsphere phantoms are to be used over a prolonged time period, their optical scattering properties should be regularly measured. Although gel wax and TiO_2_ phantoms offer much greater optical stability than those made of gel wax and silica spheres, it is still prudent to measure their optical properties before each use, as this study found these values to vary by a small amount over time.

## 5. Conclusion

Gel wax has proven to be a simple and affordable phantom matrix material, in which exogenous scatterers can easily be added. However the poor temporal stability of silica microspheres embedded within such a matrix material preclude their repeated use over time without their repeated characterisation. It is therefore recommended that TiO_2_ is used as an alternative, more stable scattering medium when the long-term scattering stability is imperative to gel wax based phantom design.
